# Supplementary material for: Exploring the influence of COVID-19 stress on mental health among international undergraduate and graduate students: A mixed-methods approach
Source: PLoS One. 2026 Feb 6;21(2):e0336446. doi: 10.1371/journal.pone.0336446 (PMC12880682; doi:10.1371/journal.pone.0336446)
Supplement: S1 Table — (DOCX) [file pone.0336446.s001.docx]

**S1 Table.** Item-Total Correlations and Cronbach’s Alpha for the Modified COVID-19 Stress Scale

| **Subscale** | **Item** | **Item-Total Correlation** |
| --- | --- | --- |
| **Danger** (α = 0.88) | D1 | 0.69 |
|  | D2 | 0.67 |
|  | D3 | 0.71 |
|  | D4 | 0.65 |
|  | D5 | 0.72 |
|  | D6 | 0.70 |
| **Socioeconomic Consequence** (α = 0.93) | SC1 | 0.84 |
|  | SC2 | 0.84 |
|  | SC3 | 0.86 |
|  | SC4 | 0.83 |
|  | SC5 | 0.73 |
|  | SC6 | 0.73 |
| **Contamination** (α = 0.91) | C1 | 0.64 |
|  | C2 | 0.85 |
|  | C3 | 0.76 |
|  | C4 | 0.84 |
|  | C5 | 0.76 |
|  | C6 | 0.68 |
| **Traumatic Stress** (α = 0.90) | TS1 | 0.75 |
|  | TS2 | 0.66 |
|  | TS3 | 0.72 |
|  | TS4 | 0.75 |
|  | TS5 | 0.80 |
|  | TS6 | 0.71 |
| **Checking** (α = 0.88) | CH1 | 0.68 |
|  | CH2 | 0.73 |
|  | CH3 | 0.63 |
|  | CH4 | 0.69 |
|  | CH5 | 0.70 |
|  | CH6 | 0.72 |

Note: These values reflect analyses using the modified COVID-19 Stress Scale (Taylor et al., 2020)
