# Supplementary material for: Exploring the influence of COVID-19 stress on mental health among international undergraduate and graduate students: A mixed-methods approach
Source: PLoS One. 2026 Feb 6;21(2):e0336446. doi: 10.1371/journal.pone.0336446 (PMC12880682; doi:10.1371/journal.pone.0336446)
Supplement: S1 Data — (ZIP) [file pone.0336446.s002.zip › Code.docx]

TITLE: International students COVID stress

DATA:

FILE IS covid.txt;

NOBSERVATIONS = 219;

VARIABLE:

NAMES ARE stress covstr anx dep age sex marr educ;

USEVARIABLES ARE stress covstr anx dep marr educ coveduc;

DEFINE:

CENTER stress covstr (GRANDMEAN);

coveduc=covstr*educ;

!ANALYSIS:

! TYPE IS GENERAL;

MODEL:

anx ON stress covstr educ coveduc;

dep ON stress covstr marr educ coveduc;

OUTPUT:SAMPSTAT RESIDUAL STDYX modindices(all,0) TECH1;
